# P-1223. Mechanistic Insights to Combating *Enterobacter cloacae* in Deep Seated Infections

**DOI:** 10.1093/ofid/ofae631.1405

**Published:** 2025-01-29

**Authors:** Rachel Gray, Elizabeth May, Ashlan Kunz Coyne

**Affiliations:** University of Kentucky College of Pharmacy, Lexington, Kentucky; University of Kentucky College of Pharmacy, Lexington, Kentucky; University of Kentucky College of Pharmacy, Lexington, Kentucky

## Abstract

**Background:**

*Enterobacter cloacae* is a formidable pathogen in deep-seated infections. Increasing prevalence of carbapenem-resistant Enterobacterales exemplifies the need for optimizing non-carbapenem therapeutics. We aimed to evaluate cefepime (FEP) and meropenem (MEM) regimens against *E. cloacae* isolates in a pharmacokinetic/pharmacodynamic (PK/PD) *ex vivo* model.

**Table 1.**
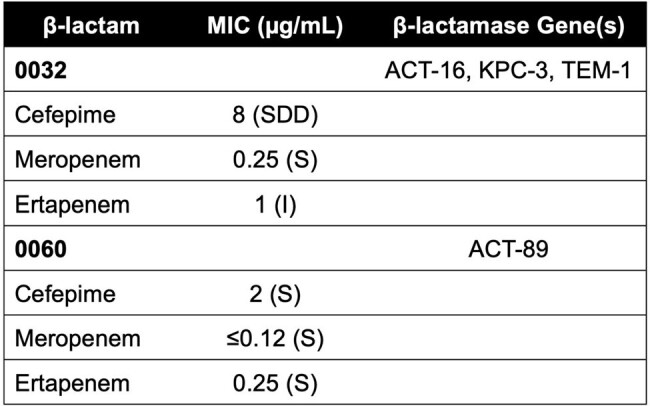
CDC Antibiotic Resistance Isolate Bank Enterobacter cloacae Strains Tested in Ex vivo Models

**Methods:**

*E. cloacae* isolates (CDC# 0032, 0060, Table 1) were evaluated in 96h simulated endocardial vegetation (SEV) high inoculum (10^9^ CFU/g) models against humanized FEP (2g q8h via 30-min and 3h infusions) and MEM (2g q8h via 30-min and 3h infusions) regimens. SEV clot and planktonic samples were removed in duplicate from each model replicate at predefined time points and evaluated for bactericidal activity (defined as a ≥ 3 log_10_ CFU/g reduction from baseline), antibiotic PK target attainment, treatment-emergent resistance, and β-lactamase induction via RT-PCR. Significant differences between regimens were assessed by ANOVA with Tukey’s post hoc modification (α=0.05).

**Table 2.**
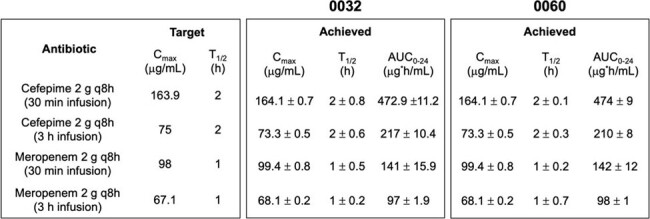
Beta-lactam PK/PD Targets and Achieved Concentrations in Ex vivo Models

**Results:**

Against *E. cloacae* 0060, MEM via 3h infusion demonstrated bactericidal activity (-Δ4.79 log_10_ CFU/g) in SEV clots and greater killing than MEM via 30-min infusion (-Δ2.6 log_10_ CFU/g; p< 0.001) (Figure 1). Both MEM 3h and 30-min infusions suppressed bacterial growth at detection limits in planktonic samples through 96h against 0060. Against *E. cloacae* 0032, neither FEP nor MEM regimens demonstrated bactericidal activity or growth suppression at 96h in SEV clots and planktonic samples, respectively (Figure 2). β-lactam PK/PD targets and achieved concentrations in *ex vivo* models are displayed in Table 2. Treatment-emergent resistance was identified in 96h samples for FEP against 0060 and MEM against 0032 (3- and 5-fold MIC increase). FEP exposure in 0060 models induced ACT-89 β-lactamase expression while FEP and MEM in 0032 models induced ACT-16, KPC-3, and TEM-1.

**Figure 1.**
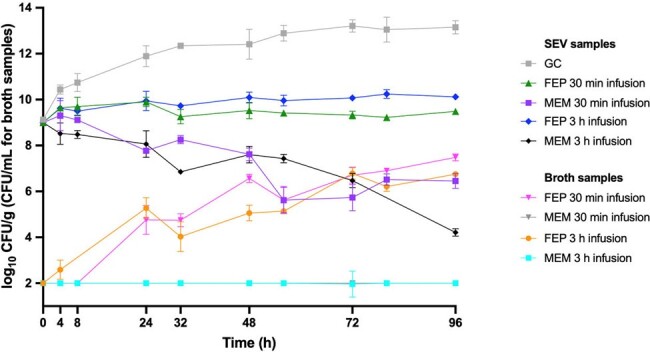
Efficacy of Beta-lactam Regimens in a SEV PK/PD Ex vivo Model Against E. cloacae Isolate 0060

**Conclusion:**

Against *E. cloacae* isolates in a PK/PD *ex vivo* model, MEM demonstrated bactericidal activity against *E. cloacae* 0060, with greater efficacy via 3h compared to 30-min infusion. However, neither FEP nor MEM showed bactericidal activity against *E. cloacae* 0032, with the emergence of treatment-resistant strains and induction of β-lactamase expression observed in some cases.

**Figure 2.**
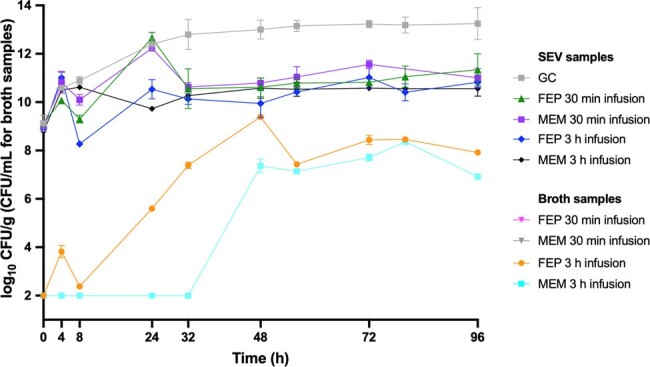
Efficacy of Beta-lactam Regimens in a SEV PK/PD Ex vivo Model Against E. cloacae Isolate 0032

**Disclosures:**

**All Authors**: No reported disclosures

